# Understanding the Improvement in Full Childhood Vaccination Coverage in Ethiopia Using Oaxaca–Blinder Decomposition Analysis

**DOI:** 10.3390/vaccines8030505

**Published:** 2020-09-04

**Authors:** Abrham Wondimu, Qi Cao, Derek Asuman, Josué Almansa, Maarten J. Postma, Marinus van Hulst

**Affiliations:** 1 Department of Pharmaceutics, School of Pharmacy, College of Medicine and Health Sciences, University of Gondar, Gondar P.O. Box 196, Ethiopia; 2 Department of Health Sciences, University Medical Center Groningen (UMCG), University of Groningen, 9713 AV Groningen, The Netherlands; groningenqi@gmail.com (Q.C.); j.almansa.ortiz@umcg.nl (J.A.); m.j.postma@rug.nl (M.J.P.); r.hulst@mzh.nl (M.v.H.); 3 Unit of Pharmaco-Therapy, Epidemiology & Economics (PTE2), Department of Pharmacy, University of Groningen, 9713 AV Groningen, The Netherlands; 4 Health Economics Unit, Medicon Village, Lund University, SE-223 81 Lund, Sweden; derek.asuman@med.lu.se; 5 Department of Economics, Econometrics & Finance, Faculty of Economics & Business, University of Groningen, 9747 AE Groningen, The Netherlands; 6 Department of Pharmacology and Therapy, Faculty of Medicine, Universitas Airlangga, Surabaya 60132, Indonesia; 7 Department of Clinical Pharmacy and Toxicology, Martini Hospital, 9728 NT Groningen, The Netherlands

**Keywords:** childhood vaccination, coverage, improvement, Ethiopia, Oaxaca–Blinder decomposition

## Abstract

In Ethiopia, full vaccination coverage among children aged 12–23 months has improved in recent decades. This study aimed to investigate drivers of the improvement in the vaccination coverage. The Oaxaca–Blinder decomposition technique was applied to identify the drivers using data from Ethiopian Demographic and Health Survey conducted in 2000 and 2016. The vaccination coverage rose from 14.3% in 2000 to 38.5% in 2016. The decomposition analysis showed that most of the rise in vaccination coverage (73.7%) resulted from the change in the effect of explanatory variables over time and other unmeasured characteristics. Muslim religion had a counteracting effect on the observed increase in vaccination coverage. The remaining 26.3% of the increase was attributed to the change in the composition of the explanatory variables between 2000 and 2016, with maternal educational level and maternal health care utilization as significant contributors. The findings highlight the need for further improvements in maternal health care utilization and educational status to maintain the momentum towards universal coverage of childhood vaccination. Targeted intervention among Muslim-dominated communities is also needed to improve the current situation. Besides which, future studies need to be conducted to identify additional potential modifiable factors.

## 1. Introduction

Vaccines are among the most cost-effective preventive health interventions in reducing childhood mortality and morbidity across the world [1]. It is estimated that childhood vaccines currently save 2–3 million lives annually and many more deaths could be averted with an elevated vaccination coverage [2]. Vaccinations can also minimize inappropriate use of antibiotics by reducing antibiotic use as a result of fewer infections among vaccinated individuals [3,4]. Vaccinations have been shown to have a significant positive effect on child growth by reducing stunting and children being underweight [5]. Beyond their impact on health, vaccines contribute to increased productivity, improved educational attainment, and economic growth [6,7,8,9].

The establishment of the Expanded Programme on Immunization (EPI) by the World Health Organization (WHO) in 1974 has resulted in substantial improvements in access to routine childhood vaccines. Global immunization coverage was less than 5% in 1974, but increased to 86% in 2018 [10]. The EPI program has been in place in Ethiopia since 1980 [11]. The program currently recommends that children in Ethiopia should receive one dose of Bacillus Calmette–Guerin (BCG) vaccine, three doses of Oral Polio Vaccine (OPV), three doses of Pentavalent vaccine (antigens against diphtheria, tetanus, pertussis, hepatitis B and Haemophilus influenza type B), three doses of pneumococcal conjugate vaccine, two doses of rotavirus vaccine and one dose of measles vaccine in their first year of life. Recently, the second dose of the measles vaccine has been included in the routine immunization schedule to be administered to the child at the age of 15 months [12,13].

Over the last two decades, Ethiopia has seen a remarkable improvement in child health, as reflected in various indicators including infant and under-five mortalities [14]. Between 2000 and 2019, the under-five mortality rate fell from 166 to 55 deaths per 1000 live births—a 67% decline. During the same period of time, the prevalence of stunting among children under the age of five decreased from 58% to 37%. The infant mortality fell to 43 deaths per 1000 live births in 2019 from 97 in 2000, which is equivalent to a 56% decline [14,15]. However, vaccine-preventable diseases remain the main causes of childhood mortality and potential deterrents to achieving the Sustainable Development Goal (SDG) target for child mortality [16,17].

Although vaccination coverage in Ethiopia is far from the goal laid out in the Global Vaccine Action Plan (GVAP), full vaccination coverage representing one dose of Bacillus Calmette–Guerin (BCG) vaccine, three doses of pentavalent vaccine (diphtheria, pertussis, tetanus, Haemophilus influenza type B and Hepatitis B combination vaccine), three doses of polio vaccine, and one dose of measles vaccine among children aged 12–23 months, increased from 14.3% in 2000 to 38.5% in 2016 [15,18]. However, little is known about the exact factors enabling this improvement in childhood vaccination coverage in Ethiopia. Therefore, this study aimed to explore the role of various determinants of the change in childhood vaccination coverage between 2000 and 2016 using the Oaxaca–Blinder decomposition analysis technique based on data from Ethiopian Demographic and Health Survey (EDHS). This approach allowed us to analyze how a particular outcome over a given time period is affected by the corresponding shift in composition and the effects of explanatory variables [19,20]. The method has previously been applied in various studies to account for the factors that drive changes in specific health variables over a certain period of time [21,22,23,24]. It is hoped that the findings of our study will make an important contribution to future policy development efforts to enhance the national coverage of childhood vaccination in Ethiopia.

## 2. Methods

### 2.1. Source of Data

This study used data from EDHS conducted in 2000 and 2016. In more than 90 countries around the world, Demographic and Health Surveys (DHSs) provide data on a variety of demographic and health issues, including maternal and child health, fertility, family planning, nutritional status, HIV/AIDS, malaria, and other household characteristics. The EDHS collected country representative data from all nine regions, two administrative cities, and urban and rural areas using a two-stage, stratified cluster sampling design, which is specified in the final EDHS reports [15,18]. Information on the child vaccination status was obtained from the recode files for children collected by the women’s questionnaires. A total of 2129 (EDHS 2000) and 2004 (EDHS 2016) children aged 12–23 months were included in our study.

### 2.2. Outcome Variables

In our study, the outcome variable was the vaccination status of children aged 12–23 months. A child was considered as ‘fully vaccinated’ if he/she received one dose of BCG vaccine, three doses of pentavalent vaccine (diphtheria, pertussis, tetanus, Haemophilus influenza type B and Hepatitis B combination vaccine), three doses of polio vaccine, and one dose of measles vaccine, and if not, the child was considered as ‘non/unvaccinated’. In accordance with the Ethiopian EPI schedule, children aged 12 to 23 months represent the youngest cohort of children who are supposed to take all vaccines recommended in the first year of life [25]. Certain vaccines, such as pneumococcal conjugate vaccine and rotavirus vaccine, which have recently been introduced in Ethiopia, have not been included in the analysis as information on their uptake is available only in the recent survey.

### 2.3. Explanatory Variables

Various demographic and health variables were included as the explanatory variables in the Oaxaca–Blinder decomposition analysis. The selection of the variables was guided by the literature and the availability of the variables in the EDHS datasets [12,26,27,28]. The following variables were therefore included in our study: mother’s age in years (15–19/20–34/35–49), mother’s and partner’s educational status (no education/primary or above), mother’s religious affiliation (Muslim/Christian and others), mother’s occupational status (employed/not employed), exposure to media (yes/no), wealth quintile (poorest/poorer/middle/richer/richest), and place of residence (rural/urban). In addition, sex of the child (male/female) and maternal health service utilization indicators including at least four antenatal care visits (yes/no) and institutional delivery (yes/no) were also included in the analysis. Moreover, administrative regions were included as explanatory variables after grouping into three groups, namely developing regions, emerging regions, and Addis Ababa and Dire Dawa. Developing regions consisted of Tigray, Amhara, Oromia, Southern Nations Nationalities and Peoples (SNNP) and Harari regions. On the other hand, Afar, Somali, Benishangul-Gumuz and Gambela regions were categorized as emerging regions based on their development profile. Addis Ababa and Dire Dawa were grouped together as urban centers.

### 2.4. Statistical Analyses

The study used descriptive statistics to show the distribution of respondents by their background characteristics. The chi-square tests were applied to compare the variables between the two surveys. Oaxaca–Blinder decomposition analysis was conducted to explore the change in full vaccination coverage in Ethiopia between 2000 and 2016. This method disaggregates the source of the change in full vaccination coverage over time into “explained” and “unexplained” components. The explained component, also referred to as an endowment or compositional effect, captures the change due to the shift in the composition of the explanatory variables over time (in our case between the two surveys). It represents the change in full vaccination coverage that would shrink when the level of the measured explanatory variable had remained the same between the two surveys given that other things were unchanged. The unexplained component (referred to as structural or coefficient effect) relates, in addition to the effect of unmeasured variables that were not included in the regression model, to the change in the effect of explanatory variables over time. It represents the change in full vaccination coverage that would persist even if the level of explanatory variables were the same between the two surveys [12,29].

Since our outcome variable is binary, a nonlinear Oaxaca–Blinder decomposition method was used [30]. Using a logit function, the probabilities of the child being fully vaccinated in 2000 (*Y*_2000_) and 2016 (*Y*_2016_) are given by

(1) $$Y_{2000}=F\left(X\beta \right)$$

(2) $$Y_{2016}=F\left(X\beta \right)$$

where *X* is a vector of explanatory variables and *β* is a vector of estimated coefficients.

Then, by using the Oaxaca–Blinder decomposition technique, the change in the mean probability of being fully vaccinated between 2000 and 2016 can be decomposed as

(3) $$\Delta Y=Y_{2016}-Y_{2000}=\;\bar{F\left(X_{2016}\beta_{2016}\right)}-\bar{F\left(X_{2000}\beta_{2000}\right)}$$

By adding and subtracting $\bar{F\left(X_{2016}\beta_{2000}\right)}$ on Equation (2) and rearranging it, the decomposition equation can be re-written as the sum of the two components.

(4) $$Y_{2016}-Y_{2000}=\left\{\bar{F\left(X_{2016}\beta_{2000}\right)}-\bar{F\left(X_{2000}\beta_{2000}\right)}\right\}+\left\{\bar{F\left(X_{2016}\beta_{2016}\right)}\;-\bar{F\left(X_{2016}\beta_{2000}\right)}\right\}$$

The first term in curly brackets, $\bar{F\left(X_{2016}\beta_{2000}\right)}\;-\bar{F\left(X_{2000}\beta_{2000}\right)}$, represents the explained or compositional component. It accounts for the change in full vaccination between 2000 and 2016 due to the change in the composition of the explanatory variables (*X*’s) between 2000 and 2016. The second term (coefficient component), $\bar{F\left(X_{2016}\beta_{2016}\right)}\;-\bar{F\left(X_{2016}\beta_{2000}\right)}$, represents the change due to variation in the regression coefficients (*β*’s) over time, which measures the change in the effect of the explanatory variables. Several previous studies have also used a nonlinear Oaxaca–Blinder decomposition technique to decompose the change or difference in the level of binary outcome variables between two groups [31,32,33,34,35,36,37,38].

The presence of multicollinearity among the explanatory variables was checked by using variance inflation factor (VIF) at a cut-off point of 10. The decomposition analysis was conducted using the *Oaxaca* package in Stata version 15.1 (StataCorp., College Station, TX, USA). All estimates were weighted to account for the complex sampling procedure used in the EDHS.

## 3. Results

### 3.1. Descriptive Statistics

A total of 2129 and 2004 children aged 12–23 months from EDHS 2000 and EDHS 2016 were included in the analysis, respectively. The distribution of explanatory variables by survey year is indicated in Table 1. The majority of mothers were rural dwellers, from the developing regions, and were aged between 20 and 34 years in both surveys. The proportion of mothers and their partners who completed at least primary education increased from 20.3% and 37.7% in 2000 to 37.3% and 52.4% in 2016, respectively. The proportion of mothers exposed to media increased from 9.5% in 2000 to 18.9% in 2016. Moreover, antenatal care utilization and institutional delivery notably increased over time. The full vaccination coverage among children aged 12–23 months rose from 14.3% to 38.5% over the inter-survey period (*p* < 0.001).

### 3.2. Oaxaca–Blinder Decomposition Analysis

A summary of Oaxaca–Blinder decomposition analysis is reported in Table 2. As shown in the table, the probability of full vaccination among children aged 12–23 months was 0.142 (95% CI: 0.122–0.162, *p* < 0.001) in 2000 and 0.394 (95% CI: 0.354–0.434, *p* < 0.001) in 2016, resulting in a statistically significant increase of 0.252 (95% CI: 0.207–0.296, *p* < 0.001). About 74% (0.186 units from the total of 0.252 units change in full vaccination, *p* < 0.001) of the increase in full vaccination coverage between 2000 and 2016 is due to the change in the effect of various explanatory variables on vaccination coverage between the two surveys and due to other unmeasured characteristics represented by the constant term (coefficient component). The remaining 26% (0.066 units, *p* < 0.001) of the increment is attributed to the change in the composition of the explanatory variables between 2000 and 2016 (compositional component).

The result of the detailed decomposition showing the contribution of the individual variables and components is presented in Table 3. The shift in the composition of maternal education and maternal health care utilization made a significant contribution to the increase in full vaccination coverage in Ethiopia between 2000 and 2016. Maternal education accounted for 0.015 (95% CI: 0.006–0.024, *p* = 0.002) unit change in full vaccination coverage between the two surveys, which is 5.9% of the total predicted change. Similarly, of the overall change, 23.2% (0.058 units, 95% CI: 0.034–0.083) is attributed to the improvement in maternal health care utilization between the two surveys. On the other hand, the most significant contributors to the unexplained portion of the increase in full vaccination coverage are Muslim religion (which contributed to −17.2% of the total change, *p* = 0.009) and the constant term (which contributed to 118.0% of the total change, *p* < 0.001).

## 4. Discussion

This study assesses the drivers of the change in full vaccination coverage among children aged 12–23 months in Ethiopia between 2000 and 2016. The decomposition analysis showed that the probability of full vaccination increased by 0.252 between the inter-survey period, of which 0.066 (26.3%) was due to the change in the composition of variables over the years (explained/compositional component). In particular, the rise in maternal health care utilization level and improvement in maternal education significantly contributed to the overall increase in full childhood vaccination in Ethiopia. In 2000, 10.5% of mothers received four or more ANC services, which increased to 33.2% in 2016. Similarly, the institutional delivery among mothers also showed a remarkable improvement, from only 5% in 2000 to 37% in 2016. The inter-survey changes in maternal health care utilization contributed to about 23% of the increase in full childhood vaccination coverage. On the other hand, the compositional change in maternal education between 2000 and 2016 accounts for 6% of the increase in vaccination coverage. Previous studies have documented ANC utilization [39,40,41], institutional delivery [42,43], and maternal educational level [44,45,46,47,48,49] as strong predictors of childhood vaccination. Therefore, this might be why the change in the level of these predictors over years has brought an increase in full childhood vaccination coverage in Ethiopia.

The majority of the change (73.7%) in vaccination coverage between 2000 and 2016 is attributed to the change in effect of the explanatory variables and other unmeasured characteristics which are not included in the model. This suggests that if the composition, and thus levels of the explanatory variables, were the same between 2000 and 2016, the probability of full vaccination among children would still have increased by a level of 0.186 (73.7% of the estimated increment) due to a change in the effect of the explanatory variables. This could be attributed to various policy measures taken in the last two decades in Ethiopia that brought structural changes that might significantly increase the propensity of child vaccination. The period coincided with the era of the Millennium Development Goals (MDGs), during which various national and international efforts were made to improve the overall health care system. Ethiopia achieved most of the health-related MDGs [50]. The Health Extension Program was introduced in Ethiopia in 2003 and immunization is one of the key components of the program. By the year 2015, about 38,000 health extension workers (HEWs) were deployed in Ethiopia [51,52]. The HEWs are expected to increase demand for different maternal and child health services among community members, including vaccination uptake through awareness-raising. The positive link between childhood immunization and health extension program in Ethiopia has been documented in a number of studies [51,53,54].

The rapid expansion of the health infrastructure could also contribute to improving the country’s immunization service. Between 2005 and 2015, for instance, the number of hospitals, health centers, and health posts rose from 88 to 241, from 690 to 3562 and from 8528 to 16,480, respectively [50].

The “reach every district” (RED) approach, which was developed by WHO and its collaborators in 2002, is another remarkable strategy introduced in most African countries, including Ethiopia, to enhance immunization coverage through application of operational strategies including (1) planning and management of resources; (2) reaching all eligible populations; (3) engaging with communities; (4) conducting supportive supervision; and (5) monitoring and using data for action [55,56]. An improvement in immunization services was noted after the implementation of this strategy [57].

Among the explanatory variables, being Muslim had a significant counteracting effect on the overall increment of childhood vaccination coverage in Ethiopia between 2000 and 2016 (−17.2%), compared to Christians and others. This underlines the need for further study to explore the role of religious affiliation in childhood vaccination status. Nonetheless, we recommend the involvement of religious leaders and targeted intervention in Muslim-dominated regions to increase the coverage of vaccination among children in Ethiopia. A previous study in 15 sub-Saharan African countries, including Ethiopia, showed that being Muslim has been associated with lower immunization rates [58].

Notably, our study revealed that the overall increment in full vaccination coverage in Ethiopia between 2000 and 2016 was significantly driven by the change in the constant term (107.5%) indicating that the increment was strongly influenced by other determinants not explicitly considered in our model. Apart from the factors included in our study, there are obviously other factors, such as cold transport and storage, vaccine supply and quality of the immunization service, which could potentially influence vaccination coverage [59,60]. Given that most of the increase in vaccination coverage between 2000 and 2016 is driven by unknown factors, it is essential to invest in future studies to discover these factors.

Methodologically speaking, this study has two main limitations. First, Oaxaca–Blinder decomposition analysis only allows the inclusion of no more than two time periods. This may hinder the disclosure of how childhood vaccination coverage changed during the intermediate stage between 2000 and 2016. Besides multiple decomposition analyses that contain pairwise comparison in each analysis, one can use multilevel logistic regression which includes the time periods as the explanatory variable [43]. Our second limitation is that the outcome variable was categorized only into two groups (i.e., full vaccination, no vaccination). This categorization is quite extreme and does not consider the child who receives part of the vaccines (i.e., not completely vaccinated). Despite these limitations, the findings from the current study have certain public health implications in relation to childhood vaccination coverage in Ethiopia. There is a need to improve maternal health care utilization (e.g., antenatal care use, institutional delivery) and increase access to maternal education in the country in order to maintain the observed positive change in vaccination coverage. It is also important to reinvigorate the structural changes that have been made in the health care sector in the country.

## 5. Conclusions

The coverage of full childhood vaccination in Ethiopia improved significantly between 2000 and 2016. The improvement in coverage is mainly driven by a change in coefficients attributed to structural changes over the years. The compositional change in maternal health care utilization and maternal education also significantly contributed to the increase in full vaccination coverage. As an important share of the increase in vaccination coverage is driven by unknown factors, future studies need to be conducted to identify additional potential modifiable factors not observed in this study.

## Figures and Tables

**Table 1:** Distribution of explanatory variables by survey year.

Variable	EDHS 2000 (*N* = 2129 ^‡^) %	EDHS 2016 (*N* = 2004 ^‡^) %	*p*-Value
Mother’s age			
<20	6.0	4.2	0.190
20–34	71.8	73.2	
35–49	22.2	22.6	
Educational level of mother			
No education	79.7	62.7	<0.001
Primary and above primary	20.3	37.3	
Mother’s employment status			
Not working	37.6	54.3	<0.001
Working	62.4	45.7	
Religion			
Muslim	30.3	39.2	0.001
Christian and Others	69.7	60.8	
Place of Residence			
Rural	89.7	88.4	0.415
Urban	10.3	11.6	
Exposure to Media			
Not exposed	90.5	81.1	<0.001
Exposed	9.5	18.9	
Antenatal care use (≥4 visits)			
No	89.5	66.8	<0.001
Yes	10.5	33.2	
Delivery place			
Home	95.3	63.4	<0.001
Health Facility	4.7	36.6	
Administrative region			
Developing regions ^§^	95.2	90.9	<0.001
Emerging regions ^§§^	3.0	6.1	
Addis Ababa & Dire Dawa ^§§§^	1.8	3.1	
Wealth quintile			
Poorest	21.4	25.2	0.434
Poorer	20.8	19.8	
Middle	21.6	22.5	
Richer	19.6	18.3	
Richest	16.6	14.4	
Sex of Child			
Male	51.8	46.2	0.018
Female	48.2	53.8	
Educational level of partner			
No education	62.3	47.6	<0.001
Primary and above primary	37.7	52.4	
Full childhood vaccination coverage	14.3	38.5	<0.001

EDHS: Ethiopian Demographic Health Survey; ^‡^ Sampling weight were applied; ^§^ Tigray, Amhara, Oromia, Southern Nations Nationalities and Peoples and Harari regions; ^§§^ Afar, Somali, Benishangul-Gumuz and Gambela regions; ^§§§^ Addis Ababa and Dire Dawa.

**Table 2:** Summary of results from Oaxaca–Blinder decomposition analysis.

	Estimate [95% CI]
Predicted full vaccination coverage for EDHS2016	0.394 [0.354, 0.434] ***
Predicted full vaccination coverage for EDHS2000	0.142 [0.122, 0.162] ***
Difference	0.252 [0.207, 0.296] ***
Due to the change in composition of the variables	0.066 [0.037, 0.096] ***
Due to the change in effect of variables and constant term	0.186 [0.134, 0.238] ***

EDHS: Ethiopian Demographic Health Survey; *** *p* < 0.001.

**Table 3:** Contribution of the change in composition of the variables (explained component) and the change in the effect of the variables and constant term (coefficient component) to the increase in full vaccination coverage in Ethiopia during 2000–2016.

Variable	Composition of the Variables ^†^		Effect of Variables and the Constant Term ^†^	
	Absolute Contribution	% Share ^††^	Absolute Contribution	% Share ^††^
Maternal age				
<20	−0.001 [−0.002, 0.001]	−0.2	0.000 [−0.005, 0.005]	0.0
20–34	0.000 [−0.001, 0.001]	−0.1	−0.003 [−0.051, 0.045]	−1.3
35–49	0.000 [−0.001, 0.000]	0.0	0.000 [−0.019, 0.018]	−0.1
Maternal education	0.015 [0.006, 0.024] **	5.9	−0.016 [−0.040, 0.007]	−6.5
Maternal employment status	−0.005 [−0.013, 0.003]	−1.9	−0.015 [−0.061, 0.031]	−6.0
Muslim religion	−0.006 [−0.013, 0.002]	−2.3	−0.043 [−0.076, −0.011] **	−17.2
Urban residence	0.000 [−0.002, 0.002]	0.2	−0.008 [−0.027, 0.012]	−3.1
Exposure to media	0.003 [−0.003, 0.008]	1.0	−0.003 [−0.018, 0.012]	−1.2
Maternal health care utilization ^‡^	0.058 [0.034, 0.083] ***	23.2	−0.003 [−0.027, 0.020]	−1.4
Region				
Developing regions ^§^	0.003 [0.001, 0.005] **	1.3	−0.043 [−0.119, 0.032]	−17.2
Emerging regions ^§§^	−0.002 [−0.003, 0.000] *	−0.7	0.002 [−0.002, 0.006]	0.9
Addis Ababa and Dire Dawa ^§§§^	0.002 [0.001, 0.003] **	0.7	0.000 [−0.003, 0.002]	−0.1
Wealth	−0.003 [−0.009, 0.003]	−1.1	0.001 [−0.007, 0.009]	0.4
Sex of Child	0.000 [−0.002, 0.002]	−0.1	0.023 [−0.016, 0.062]	9.0
Partners educational level	0.001 [−0.006, 0.008]	0.4	−0.001 [−0.038, 0.035]	−0.6
Constant	-	-	0.297 [0.162, 0.433] ***	118.0

^†^ the negative sign indicates that the variable has a counteracting effect on the observed change in vaccination coverage; ^††^ calculated by dividing the absolute change by the overall difference (0.252); ^‡^ Antenatal care utilization and institutional delivery; ^§^ Tigray, Amhara, Oromia, Southern Nations Nationalities and Peoples and Harari regions; ^§§^ Afar, Somali, Benishangul-Gumuz and Gambela regions; ^§§§^ Addis Ababa and Dire Dawa; * *p* < 0.05; ** *p* < 0.01; *** *p* < 0.001.
